# Human activity recognition of children with wearable devices using LightGBM machine learning

**DOI:** 10.1038/s41598-022-09521-1

**Published:** 2022-03-31

**Authors:** Gábor Csizmadia, Krisztina Liszkai-Peres, Bence Ferdinandy, Ádám Miklósi, Veronika Konok

**Affiliations:** 1 Department of Ethology, Eötvös Loránd University, Budapest, Hungary; 2 MTA-ELTE Comparative Ethology Research Group, Budapest, Hungary; 3 Doctoral School of Psychology, Eötvös Loránd University, Budapest, Hungary; 4 Institute of Psychology, Eötvös Loránd University, Budapest, Hungary

**Keywords:** Psychology, Human behaviour

## Abstract

Human activity recognition (HAR) using machine learning (ML) methods has been a continuously developed method for collecting and analyzing large amounts of human behavioral data using special wearable sensors in the past decade. Our main goal was to find a reliable method that could automatically detect various playful and daily routine activities in children. We defined 40 activities for ML recognition, and we collected activity motion data by means of wearable smartwatches with a special SensKid software. We analyzed the data of 34 children (19 girls, 15 boys; age range: 6.59–8.38; median age = 7.47). All children were typically developing first graders from three elementary schools. The activity recognition was a binary classification task which was evaluated with a Light Gradient Boosted Machine (LGBM) learning algorithm, a decision tree based method with a threefold cross validation. We used the sliding window technique during the signal processing, and we aimed at finding the best window size for the analysis of each behavior element to achieve the most effective settings. Seventeen activities out of 40 were successfully recognized with AUC values above 0.8. The window size had no significant effect. In summary, the LGBM is a very promising solution for HAR. In line with previous findings, our results provide a firm basis for a more precise and effective recognition system that can make human behavioral analysis faster and more objective.

## Introduction

In behavioural sciences, the objective and quantifiable measurement of behavior is a fundamental requirement for conducting research. For this purpose, video-based or live behavior coding is a frequently used method. However, manual coding (i.e. coding by human observers based on visual inspection) is time-consuming and can have subjective components^1^.

Body movements are among the main measurable components of behavior. Therefore, the use of motion sensor devices, such as accelerometers and gyroscopes, can help behavioral scientists to automatically measure behavior, both in studies on animals^2,3^ and humans^4,5^. These devices can supply researchers with a large amount of objective and quantitative data based on the three spatial dimensions. This way, data collection can be conducted with more individuals in parallel, and over a longer period of time because these devices can be used in a wide range of environments (not only in the laboratory). This may help to solve statistical problems related to low sample size (and consequences e.g. low statistical power, not representative sample).

However, such data (big data) requires specific analytic methods. Machine learning algorithms can be used to train models that are able to automatically identify predetermined behavior categories (supervised learning^6^). This can improve behavior measurement and make it more objective than the visual inspection of behavior by human observers.

In humans, movement sensors are increasingly used in different settings, such as the entertaining or healthcare industry. Smartwatches with accelerometer and gyroscope can derive general health related parameters such as total step counts, and people often use this function for maintaining regular physical activity. These devices can be used also by healthcare professionals, for e.g. assisting rehabilitation^7,8^, monitoring physical activity in specific patient groups^9,10^, or monitoring symptoms of movement disorders (e.g. essential tremor or motor symptoms of Parkinson’s disease; for a review see^11^).

Sedentary lifestyle is increasingly frequent in children which is partly attributed to the excessive usage of digital devices^12^. This may explain why the prevalence of obesity, diabetes and other related health problems is increasing in childhood^13,14^. However, children can be especially motivated by games, and the ubiquity of smart mobile devices makes it possible to use these devices for exergaming and facilitating children to do physical activity. An additional advantage of mobile devices is that they can be used anywhere, therefore, outdoor activities can be also facilitated by them, and geolocation data can be also exploited for the game experience^15^. If machine learning based feedback can be included in a game, it may become more motivating and entertaining and can also improve motor skills (for a review see^16^).

Another potential application area for motion sensors with machine learning in healthcare is the identification of symptoms of movement disorders or neurological diseases, which can aid either in the establishment of a diagnosis^17^ or in the management of symptoms^18,19^. This method could help in diagnosing mental disorders which have no biomarker and thus, straightforward diagnosis is problematic. For example, activity-based automatic detection has been increasingly used in case of neurocognitive disorders, like autism spectrum disorder and attention-deficit hyperactivity disorder^20^. These developmental disorders start in infancy but are often diagnosed only in pre-school or school years, while earlier diagnosis leads to better prognosis^21^. Therefore, the usage of motion sensor data with machine learning has the potential to predict developmental disorders at an earlier age than usual^22^. Additionally, this method can also aid in managing symptoms that present a burden to the affected individuals and their family^23,24^.

In sum, wearable motion sensors can provide an objective research tool and a useful healthcare aid in prevention, diagnosis, and treatment. However, more research is needed on how automatic detection of activity can be optimally carried out. We propose a method with which children’s activity can be assessed and detected automatically. This way game applications can be improved which facilitate physical activity, predict neurodevelopmental disorders and/or improve motor skills in children with motor problems.

We developed a wearable system and an activity test battery for children and assumed that we can successfully identify activities by the means of machine learning models. In contrast to other approaches^4^, we aimed at applying and detecting complex (playful and everyday) activities which can form the base of a game application.

As the time spent on using mobile devices, especially playing games, increases in the school age^25^ and many children get diagnosis of developmental disorders at the beginning of elementary school^26^, both the development of game applications facilitating physical activity and the training of machine learning models to predict developmental disorders would be feasible to carry out in this age group. Therefore, in the present study we focused on 6–8-years-old children.

ML methods in Human Activity Recognition (HAR) rely on two main approaches regarding the pre-processing of data before the learning task, one is based on derivative parameters or features, and the other one works on raw data. The first one requires the segmentation of the data and the extraction of derived features. On these data mostly decision tree-based classifications are used. The second one, is the deep learning method, which automatically learns the required feature representation directly from the raw data using e.g. convolutional neural networks^27^.

The first step of any ML method is *segmentation*. i.e. dissecting the time series into smaller segments. One of three different windowing techniques are usually used to determine these segments and recognition accuracy may be affected by them and by the length of the window: (i) sliding window technique where signals are divided into equal segments using a sliding fix-length window; (ii) event-defined window techniques, where pre-processing is necessary to locate specific events, which are further used to define successive data partitioning and (iii) activity-defined window technique where data partitioning is based on the detection of activity changes. These windows may (but not necessarily) overlap, and the degree of the overlap may also affect the ML performance^28^. The sliding window approach is well-suited to real-time applications since it does not require pre-processing.

The second step of many ML methods is the *feature extraction* the aim of which is to get the most effective features from the obtained raw segments. Time domain features include mean, median, variance skewness, kurtosis, range etc. Peak frequency, peak power and spectral power on specific frequency bands and spectral entropy are generally included in the frequency-domain features. Feature extraction is crucial in any HAR targeted solution since the features used primarily determine the overall system accuracy. One widely used approach is to rely on various arbitrarily chosen measures of the raw data and then to find the most effective combination of these features (these may range from a few to many hundreds).

After segmentation and feature extraction, various classification algorithms are applied to each window, and the recognition process (decision tree based methods, most frequently boosted variants) runs on the derivative features.

There is no unified standpoint about which ML methods (including classification method, or preprocessing method) yield the best success. The choice of the classifier method is the most important parameter, followed by the segmentation method, window size and finally sampling frequency^29^. The picture is even more complex because there is also a correlation between the number of features and the window size^30^.

Therefore, our aim was to find the best machine learning method and parameters that delivers the best performance in the recognition process, is fast and effective and can also run on different types smartphones or smartwatches, so it is usable in typical life situations. Further, we aimed also to produce guidelines for other similar HAR projects targeting children's behavior.

## Results

The overall (mean) accuracy of the classification was 0.95 ± 0.04 (M ± SD) and the AUC was 0.76 ± 0.15. The performance of the LGBM model regarding the recognition of a specific activity varied from AUC = 0.5 (Shoe_off_same, Sock_off_other) to AUC = 0.98 (Hopscotch) (Table 1).

**Table 1:** The Budapest activity test battery (BATB) and performance of the machine learning methods.

Activity name	Description	Sequence	Activity type	Accuracy	AUC	Num pos	Pos %
Hopscotch	Alternating hopping on single leg and double leg on a hopscotch ground	One jump (from bouncing off till arriving)	Playful	0.9773	0.9871	868	3.97
Ball	Throwing and catching a ball	From preparing to throw (hand rising) till catching the ball	Everyday	0.9813	0.9569	556	2.54
Goliath	Walking on the toes, hands stretched upwards	From the first step till the last step; if child stops coding is paused	Playful	0.9769	0.9447	765	3.50
Drawing	Drawing with a pencil on a paper	As the pencil touches the paper till lifting up the pencil	Playful	0.9266	0.9429	2656	12.15
Crab	Crawling backward (hands stretched, legs bent, chest upwards)	From the first step till the last step; if child stops coding is paused	Playful	0.9649	0.9396	880	4.03
Swimming	Lying on stomach, hands moving around like swimming	From the first hand movement till end of the last	Playful	0.9740	0.9380	1037	4.74
Spider	Crawling forward (hands stretched, legs bent, chest upwards)	From the first step till the last step; if child stops coding is paused	Playful	0.9573	0.9314	947	4.33
Seal	Legs and hips on the ground, hands stretched, moving only by using hands	From the first step till the last step; if child stops coding is paused	Playful	0.9461	0.9159	902	4.13
Building blocks	Building a tower from 5 building blocks (coding only when the action is done with the hand smartwatch on it, and only if building a horizontal tower)	From reaching towards a cube till putting it onto another cube	Everyday	0.9329	0.9057	1773	8.11
Bear	Crawling forward (hands stretched, legs bent, chest downwards)	From the first step till the last step; if child stops coding is paused	Playful	0.9588	0.9020	787	3.60
Light off	Turning off the light, then releasing hands	From lifting hand till the hand is next to the body in the start position	Everyday	0.9820	0.8984	359	1.64

The table was sorted by AUC with the highest recognition figures at the top. Num pos is the number of occurrences of a given activity in the dataset.

**Figure 1:**
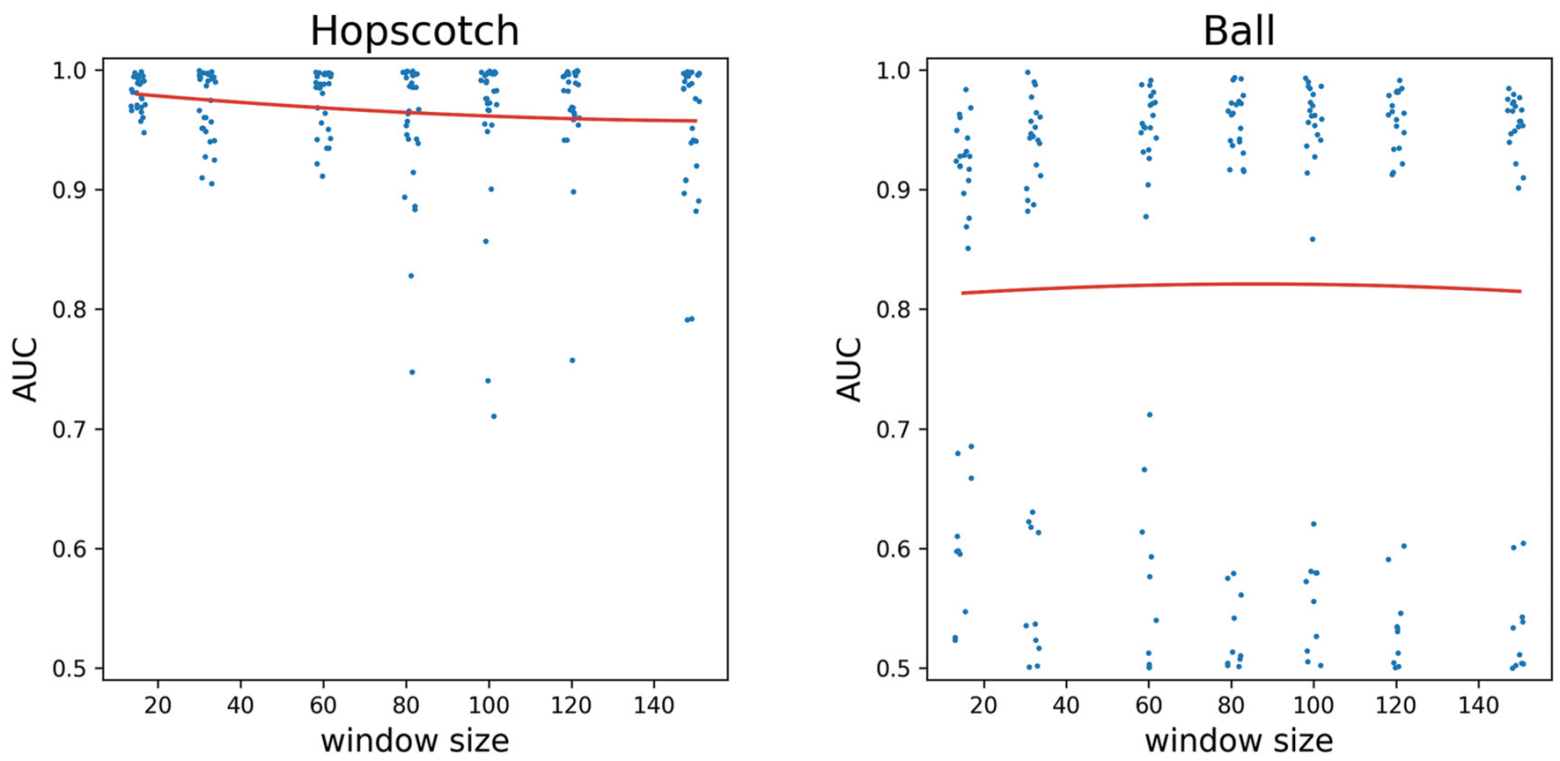
Playful activities with the highest AUC values, AUC values as a function of the window size. Quadratic curves fitted (Hopscotch: adj. R^2^ = 0.240, Ball: adj. R^2^ = 0.044). A small random jitter was added to the window sizes for better visibility.

**Figure 2:**
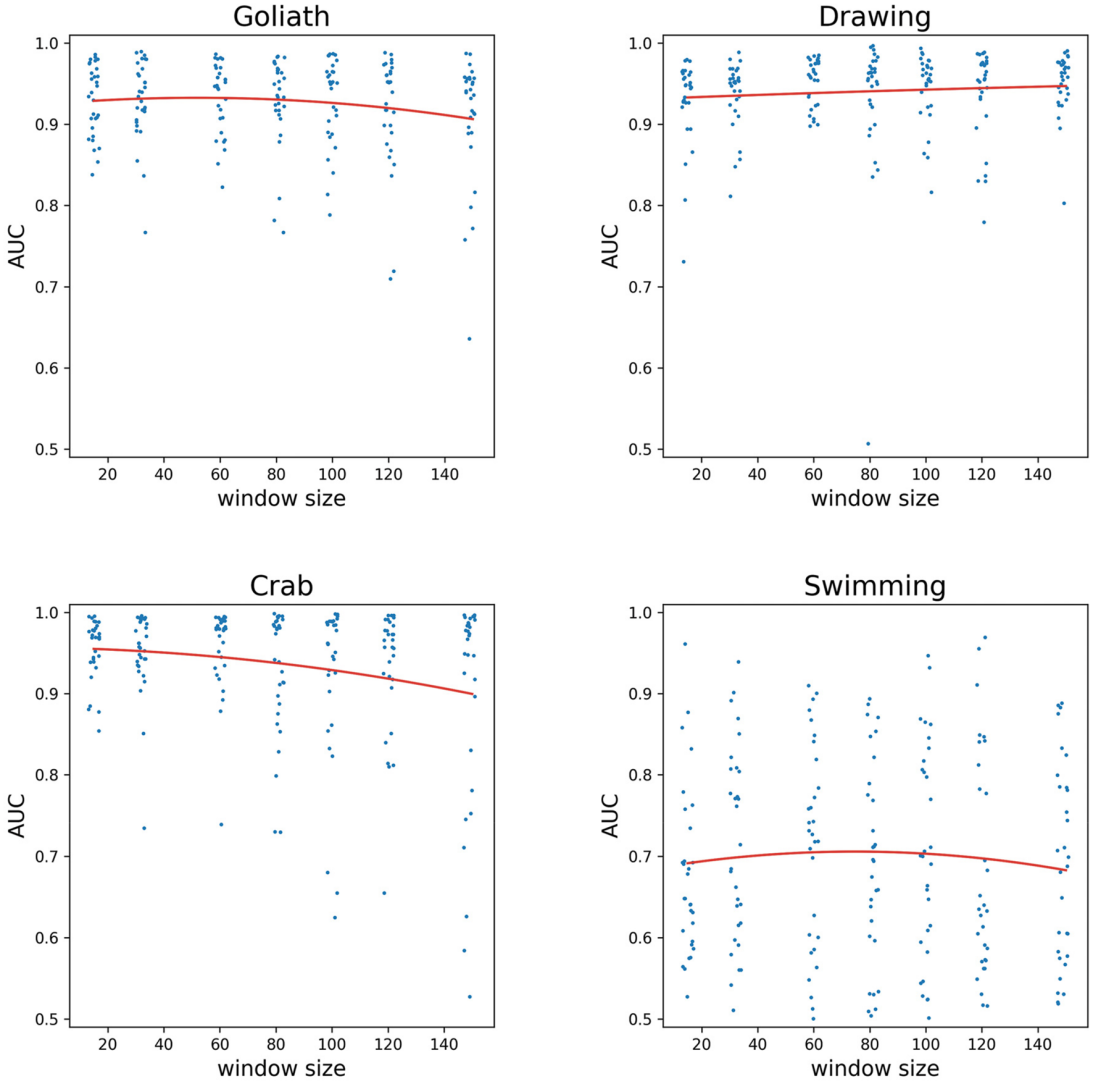
Playful activities with the highest AUC values, AUC values as a function of the window size. Quadratic curves fitted (Goliath: adj. R^2^ = 0.007, Drawing: adj. R^2^ = 0.074, Crab: adj. R^2^ = 0.084, Swimming: adj. R^2^ = 0.026). A small random jitter was added to the window sizes for better visibility.

**Figure 3:**
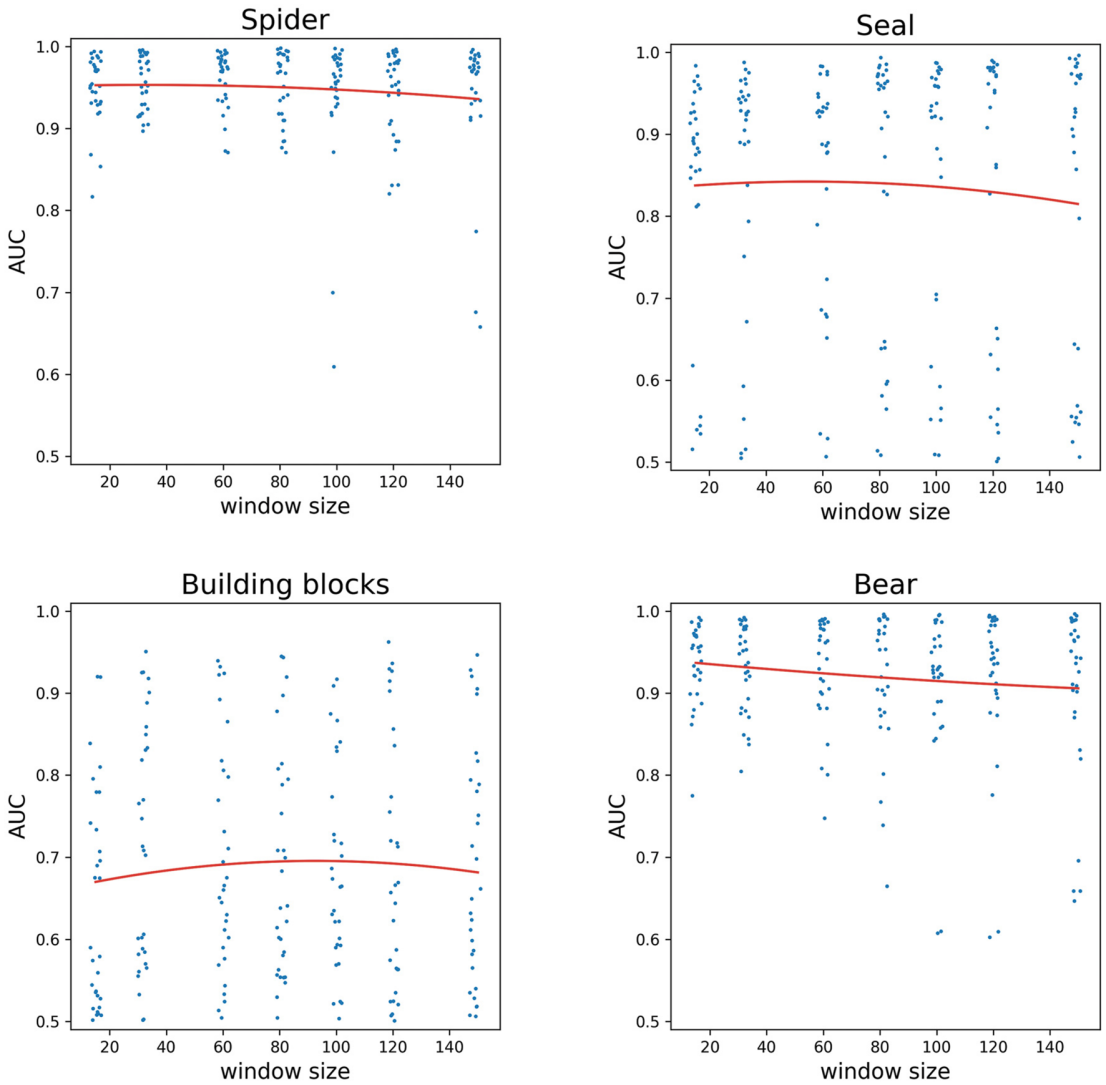
Playful activities with the highest AUC values, AUC values as a function of the window size. Quadratic curves fitted (Spider: adj. R^2^ = 0.091, Seal: adj. R^2^ = 0.327, Building blocks: adj. R^2^ = 0.036, Bear: adj. R^2^ = 0.115). A small random jitter was added to the window sizes for better visibility.

As the underlying data is inherently imbalanced, the accuracy measures could be misleading, but we provided them only for comparison with similar data in the literature, so we favour AUC as the main performance indicator. The recognition was the highest (AUC > 0.9) in the case of Hopscotch, Ball, Goliath, Drawing, Crab, Swimming, Spider, Seal, Building blocks and Bear (Figs. 1, 2, 3).

**Figure 4:**
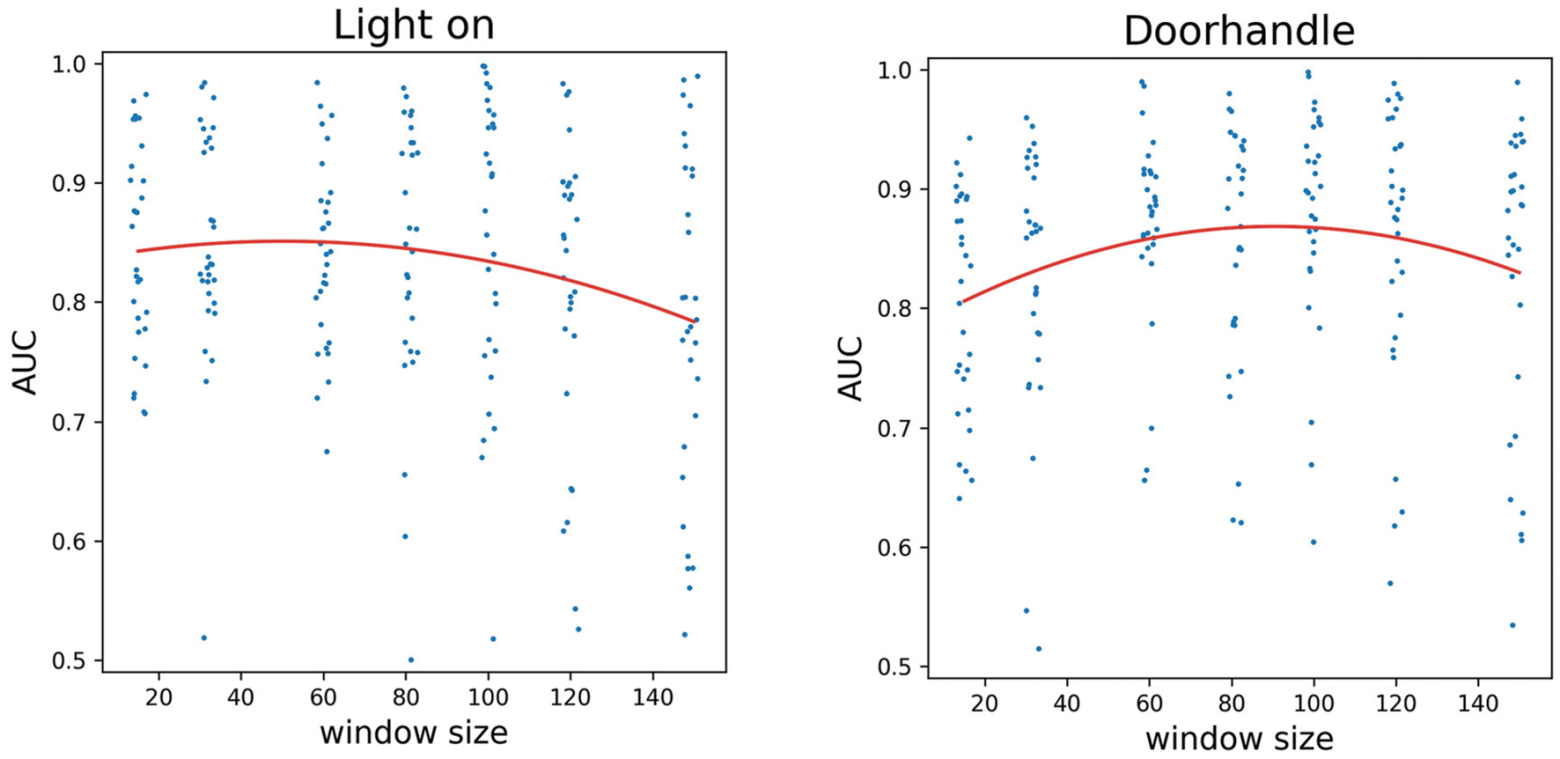
The AUC values of two everyday activities as a function of the window sizes and the quadratic curves fitted (Light on: adj. R^2^ = 0.138, Doorhandle: adj. R^2^ = 0.088) for the two activities with the highest R^2^. A small random jitter was added to the window sizes for better visibility.

To analyse whether there is an optimal window size in terms of AUC, we fitted a quadratic regression using the lm function of R, version 3.6.3. on the AUC—window size values of each activity. A quadratic curve allows for having a local maximum, enabling the investigations of the optimum. We defined the effect size as relevant if due to window size change an AUC change of 0.05 can be achieved within the 15–149 range explored. We assessed adj. R^2^ values of the regression for goodness of fit. The highest value of R^2^ was 0.327 for “Seal” (effect size = 0.027), while the highest effect size was 0.156 for Rabbit (R^2^ = 0.069, see Fig. 2), with the effect size mostly due to the linear term in the model, that is, no local maxima was found. “Door handle” is the sole activity where both measures were appreciable (R^2^ = 0.092, effect size = 0.062), while for most activities, both measures were low (see Fig. 4 and Tables 1, 2, 3). Thus, for all but one activity the applied window size did not influence the AUC values.

**Table 2:** The Budapest activity test battery (BATB) and performance of the machine learning methods.

Activity name	Description	Sequence	Activity type	Accuracy	AUC	Num pos	Pos %
Dwarf	Walking in squat position	From the first step till the last step; if child stops coding is paused	Playful	0.9512	0.8838	1113	5.09
Rabbit	Legs between hands, first moving forward hands, then jumping with legs	Begins with hand stretching, ends with completing the jump	Playful	0.9790	0.8711	362	1.66
Book	Turning pages in a book	From grasping a page till releasing it	Everyday	0.9571	0.8667	750	3.43
Nose	Touch nose	From reaching towards nose till releasing it	Everyday	0.9797	0.8576	503	2.30
Light on	Turning on the light, then releasing hands	From lifting hand till the hand is next to the body in the start position	Everyday	0.9800	0.8558	375	1.72
Door handle	Grabbing door handle, pushing down, releasing hands	From lifting hand till releasing the door handle	Everyday	0.9686	0.8451	645	2.95
Peck	Peck the skin on the back of the hand	From reaching towards the hand till releasing it	Everyday	0.9710	0.7892	535	2.45
Frog	Jumping with open legs, hands in the air beside the body	From moving upward till arriving to the lowest point	Playful	0.9780	0.7781	346	1.58
Glass grabbing	Grabbing a glass	From reaching glass till grabbing	Everyday	0.9828	0.7696	268	1.23
Pudding eat	Eating a pudding	One mouthful, from putting spoon into the mouth till moving it away from mouth	Everyday	0.9598	0.7678	836	3.82
Clapping	Clapping hands	From approximating hands till start position	Everyday	0.9825	0.7577	208	0.95
Drinking	Drinking from a glass	From touching mouth with the glass till releasing it	Everyday	0.9630	0.7473	178	0.81
Glass lifting	Lifting a glass	From grabbing till lifting the upmost point	Everyday	0.9802	0.6961	216	0.99
Sock on (same side)	Put on socks	From grasping a sock till the sock is on the foot	Everyday	0.9752	0.6889	512	2.34
Sock on (other side)	Put on socks	From grasping a sock till the sock is on the foot	Everyday	0.9697	0.6863	462	2.11

The table was sorted by AUC with the highest recognition figures at the top. Num pos is the number of occurrences of a given activity in the dataset.

**Table 3:** The Budapest activity test battery (BATB) and performance of the machine learning methods.

Activity name	Description	Sequence	Activity type	Accuracy	AUC	Num pos	Pos %
Toothbrush (other)	Put toothpaste on to the toothbrush	From grabbing toothbrush and toothpaste till finishing putting toothpaste on the toothbrush	Everyday	0.9720	0.6855	371	1.69
Hand wash	Washing hands	From rubbing hands till hands are under water	Everyday	0.9814	0.6849	285	1.30
Snack eat	Eating a snack	One bite, from putting snack into the mouth till moving away from mouth	Everyday	0.9677	0.6587	288	1.32
Knee (same)	Touch knee	From reaching towards knee till releasing it	Everyday	0.9762	0.6478	227	1.04
Knee (other)	Touch knee	From reaching towards knee till releasing it	Everyday	0.9171	0.6161	134	0.61
Shoe on (same side)	Put on shoes	From grasping a shoe till the shoe is on the foot	Everyday	0.9734	0.6139	417	1.91
Pray	Hands together in pray style	From approximating hands till releasing them	Everyday	0.9740	0.5993	389	1.78
Toothbrush (same)	Put toothpaste on to the toothbrush (same: pushing toothpaste with the hand smartwatch on it)	From grabbing toothbrush and toothpaste till finishing putting toothpaste on the toothbrush	Everyday	0.9534	0.5637	226	1.03
Glass to mouth	Lifting glass to the mouth	From lifting glass to the mouth till it touches the mouth	Everyday	0.7987	0.5631	31	0.14
Shoe off (other side)	Take off shoes, opposite side of the body relative to the hand with the smartwatch	From grasping a shoe till the shoe is off the foot	Everyday	0.8706	0.5383	53	0.24
Shoe on (other side)	Put on shoes	From grasping a shoe till the shoe is on the foot	Everyday	0.9387	0.5366	247	1.13
Sock off (same side)	Take off socks	From grasping a sock till the sock is off the foot	Everyday	0.9280	0.5110	167	0.76
Sock off (other side)	Take off socks	From grasping a sock till the sock is off the foot	Everyday	0.8481	0.5	115	0.53
Shoe off (same side)	Take off shoes, same side of the body relative to the hand with the smartwatch	From grasping a shoe till the shoe is off the foot	Everyday	0.8130	0.5	70	0.32

The table was sorted by AUC with the highest recognition figures at the top. Num pos is the number of occurrences of a given activity in the dataset.

## Discussion

Our main goal was to introduce a ML method which is able to recognize various playful and everyday activities for rapid automated analysis in children. Overall, our method of data pre-processing and the applied LGBM algorithm is successful regarding the recognition performance. Our mean accuracy (0.95) is in the top range of other similar HAR machine learning results (see summary Table 1 in^31^) When evaluating our results, it should be noted that we used only one movement sensor located on the wrist of the children.

Although the accuracy values were excellent, we also examined AUC values because those are more robust indicators of recognition success (e.g. accuracy is high even when not only true positives, but false positives are high). We obtained at least acceptable AUC values for all activities, and very good values for the majority of them, e.g. seventeen activities were recognized with AUC values above 0.8. Activities with lower AUC values are those with lower sample size (see Table 1, last column for the occurrence of the activities). Therefore, larger sample might increase the recognition rate of these activities.

We achieved the highest AUC values mainly for the playful activities. Besides that these were also among the activities most frequently carried out, but it is also possible that the LGBM method performs better in the case of complex activities that involve several body parts, with characterized sensor data patterns^32^.

The recognition of everyday activities was in the lower segment of the performance list. One possible explanation (besides lower sample size) is that some of these actions are executed very similarly in 3D space. For example, praying and clapping with the hands are very similar actions when measured by one sensor on the wrist that mainly differ in their speed of execution. This kind of problem, undeniably, could be addressed by using different feature extraction methods for various activities and optimising for unique features from the best performed IMU signals, but our intention was to develop a widely usable method which could be adopted for any kind of activity in the future. Another explanation could be that some everyday activities (e.g. taking socks on or off) could be executed variably resulting in large inter-individual differences. Such variation may make it impossible for the algorithm to find a common pattern in the feature set.

No systematic effect of the window size (window size 15 (0.3 s) to 149 (3 s)) on the AUC was revealed, suggesting that it either does not exist, or the sample size and range of values was insufficient to detect such an effect. Awais et al^33^ reported similar observations with no or little effect of the window size when they compared various experiments on HAR datasets. In contrast, Banos et al.^30^ reported a significant drop in the accuracy when they examined the effect of the window size ranging from 0.25 s to 7 s in steps of 0.25 s. Intervals below 1 s resulted in lower accuracy values. Based on our data, window size optimization has an effect only when the recognition performance is in the middle range, not too low or not too high. Both scenarios push the variance toward extreme values, so the outcome is masked by this side effect. Importantly, we used subject-independent cross-validation (CV), while window size effect was reported in studies applying a subject-dependent CV^30^. In the latter case individual differences do not mask the effect of window sizes. This is especially true for overlapping windows, which we used in this study, in case of which the performance difference between subject-dependent CV and subject independent CV increases with the window size. Dehgani et al^28^ also reported that there was no window size effect in case of subject-independent CV.

The categorization performance is better at higher AUC figures, ranging from 0.5 as minimum value to 1.0 as the maximum. However, the closer AUC is to the maximum value the lower is the variance of the AUC and as a consequence the window size has lower impact on the performance.

Our results enable the development of game applications which improve motor skills as the ML model integrated into the Senskid system is able to give feedback to the user about the accuracy of movement execution. At least the 17 movement types can be used in such applications as these were identified with very high AUC values. We achieved the highest AUC values mainly for the playful activities which children frequently execute during preschool or school activities or physical training (e.g. crab and spider crawling, bear walking, etc.). Therefore, our activity battery can be easily applied in preschools and schools with the help of the teachers. Game applications relying on ML algorithms which request and measure these activities could be used in such context to facilitate and improve movement, or predict motor or neurocognitive problems. Although some of the everyday activities were less successfully recognized in the present study, this could be improved by increasing sample size of the collected data. Many children with motor/neurocognitive problems^34^ have difficulties with performing everyday activities, thus, there is a huge need for gamified technological solutions which improve their motor skills.

## Methods

### Participants

Thirty-nine children participated in the study (22 girls, 17 boys; age range: 6.59–8.38; median age = 7.45), but the data of 5 children was not used in the analysis due to technical problems (either the movement data or the video recording was lost). Thus, finally we analyzed the data of 34 children (19 girls; 15 boys age range: 6.59–8.38; median age = 7.47). All children were typically developing first graders from three elementary schools (see Sect. 1.3). The parents of all participants gave their written informed consent to the study.

### Materials

#### Budapest activity test battery (BATB)

The Budapest Activity Test Battery (BATB) was developed by our research group, and it contains complex activities (the activities and their definitions are presented in Tables 1, 2, 3, that are executable by 6–8-year-old children. The selection criteria for the activities were that they should be motivating enough for the children to be integrated later into a game application, they should improve different motor skills after regular practice (fine and gross motor activities, arm-leg coordination, cross-side and same-side activities), and they could be either shown by a character on the mobile/tablet’s screen or explained (asked) verbally. Therefore, two main types of activities were included: playful, entertaining (mostly reproducing or mimicking movements of animals) activities that the child would be motivated to perform, and everyday activities that (being part of a child’s everyday routine) the parent would be motivated to ask from the child (e.g. taking off the shoes, washing hands).

We used different instructional methods for the playful and everyday activities. Playful activities (N = 10) were shown by the experimenter, and children were asked to imitate them. In contrast, children were instructed verbally to perform everyday activities (N = 24). If they were not able to carry them out, then the experimenter demonstrated the actual activity. The children were asked to repeat each of them 5 times in order to obtain more data. Some of the activities required some equipment e.g. ball, book, building blocks, glass, spoon, snack, toothbrush, toothpaste.

Playful activities (except for hopscotch) were repetitive movement sequences: the same movement elements were repeated over a predetermined distance (e.g. crawling like a crab across the room). Everyday activities were single (not repetitive) action sequences (their units were functional on their own, e.g. throwing up a ball, grabbing the door handle). At the end of the data collection the number of the activities varied from 31 to 2656 (number of positive, ground truth).

#### Device and software

The data collection equipment consisted of two devices, one sensor device (smartwatch) and another one (smartphone) for controlling the sensor device and managing sensor data, both running SensKid software. We used Apple Watch as sensor device which is a commercially available Apple product, regarding product details, please see https://www.apple.com/.

SensKid software is a member of the SensX software family, which is under development and not yet commercially available. The sensor device contained a 9-axis motion sensor (3-axis gyroscope + 3-axis accelerometer + 3-axis magnetometer) and samples data at 50 Hz (50 sample/sec). Each sample data point contained 3 dimensional parameters of the device except attitude which had 4 dimensions (9 + 4). During the experimental session the sensor device processed and stored the gyroscope and accelerometer data in real time. At the end of the session the processed sensor data was sent to the measurement device connected via Bluetooth that in parallel recorded the session on video, then transferred the data and the video to our network servers. The synchronisation of the raw data and the video was made automatically by the SensKid software.

#### Procedure

Data collection took place in classrooms/gyms of three elementary schools (Kispesti Vass Lajos Általános Iskola: N = 8; Virányos Általános Iskola: N = 12; Terézvárosi Két Tannyelvű Általános Iskola: N = 19). The school psychologist/ class teacher was contacted first directly via email, then, an informed consent was asked from the director of the school. The class teacher helped us in contacting the parents to obtain their informed consents. All methods were performed in accordance with the relevant guidelines and regulations, and all the experiment protocol for involving humans was in accordance to 2018 Declaration of Helsinki.

Children were tested in groups of 2 or 3 in the presence of their teacher. The experimenter informed the child about what would happen in the test and put the sensor device on the wrist of the child, on the child’s dominant hand (which was determined by offering a high five to the child and checking which hand he or she used spontaneously). The experimenter then set the connection between the smartwatch and the smartphone and launched the recording.

The order of the activities was not fixed, it was determined ad hoc, based on (1) what the child wanted to perform, (2) how exhaustive the activity was (e.g. after 4–5 playful activities, children got tired so we changed to a less exhaustive everyday activity), (3) how much space was available for the activities. Additionally, most children did not perform all activities because of time limitation, exhaustion or other personal reasons (e.g. the child did not want to or was not allowed to perform something, e.g. eating the snack). Children were asked to repeat an activity two or five times (two times in case of longer sequences, like bear walk or frog jump, and five times in case of shorter activities like switching the light on/off). The total number of the collected samples pre activity is reported in Tables 1, 2, 3.

The study was approved by the Unified Psychological Research Ethical Committee (EPKEB; reference number: 2019/18).

#### Video coding

For video coding we used Solomon Coder (© András Péter). The coding protocol included definitions of the 40 activities and the length of an activity sequence (bout length: start—endpoint, without interruption). Per definition we used the number of positives as the number of occurrences of a given activity, which means the number of bouts, activity running ongoing without being interrupted by any other activities. For the activities and their definitions, see Tables 1, 2, 3. Video recordings were coded by five coders. Video recordings were manually synchronized to the inertial data. All coders were trained using a standardised protocol of the department, and inter-coder reliability analyses were performed during training to ensure consistent labelling.

#### Data analysis

We chose LGBM for the categorisation (LGBM 3.1.1, https://pypi.org/project/lightgbm/), because our previous research (submitted) on other datasets showed that LGBM did just deliver the best performance, but it significantly over-performed other boosting methods (e.g. XGBM) in speed and computational efforts (1.2 h vs 8.5 h per iteration).

The hyperparameters were set to default, except for that we used the ‘unbalance = true’ parameter, as our dataset is unbalanced as it’s expected. To evaluate the machine learning model, we separated the data to independent data sets for training, validating and testing. This has been carried out by k-fold cross-validation (CV). In k-fold CV, the training data is randomly partitioned in k equal subsets. The model is then trained on k − 1 subsets, and the remaining one is used for validation. We used a threefold cross validation, splitting the dataset 25 + 25% for train and 25% for validation group. We used the validation group to tune the hyper-parameters and check the overfitting. Then all the remaining 25% of the dataset made up the test group. We did the cross-validation per child, so the training and test sets did not contain the data of the same subject. Therefore, this method is referred to as subject-independent CV.

One of the most important statistical assumptions for ML processes is that samples are independent and identically distributed, that is, all the data points are sampled independently from the same distribution. However, samples drawn from the same subject are most likely not independent. This means that the similarity of samples drawn from the same participants is likely to be higher than that of samples drawn from different participants (see also^35^).

This kind of bias of k-fold CV may overestimate the performance of categorization. This problem of k-fold CV is more serious when it is used with overlapping sliding windows, as in our experiment, because these overlaps between adjacent windows are another source of unwanted dependency between data points. To address these issues, the training and testing sets should be split by participants. According to this method, which is known as subject-independent CV, in each iteration the model is trained on all the participants except those, which are used for testing. In our case, we separated the participants into 3-folds using 2-folds for training and one-fold for testing.

We used the dynamic overlapping sliding window technique for segmentation of the data with 5 sampling unit shifts. Sliding window sizes of 15, 32, 60, 81,100, 149 sample points (0.3, 06, 1.2, 1.6, 2.0, 2.9 s) was considered for feature computation; this provided sufficient temporal resolution of activity and was short enough to capture bouts of activities with the shortest duration. Successive windows had an overlap of 5 sample points. Windows containing transitions between different activities were labelled as the activity at the end of the bout. Thus each window contained activity data corresponding to exactly one video-labelled activity.

As any multi-class problem could be built up from binary classifications, we decided to use separate binary models, not one multi-label, one for each activity, and comparing the positive class to the remaining 39 activities. We ran 20 iterations per window size per fold, 60 total runs for every activity and calculated the weighted AUC value (0.5–1.0) of the run of the activity as the indicator of recognition success. We used the feature set as published earlier in^35^.

## Conclusion

In summary, we collected activity motion data with a special SensKid software by means of wearable smartwatches on the children’s wrist, asking them to show various kinds of daily routine or playful activities. We analysed the data of 34 children who were typically developing first graders from three elementary schools. Our aim was to build a machine learning model which could recognize these activities.

Light Gradient Boosted Machine (LGBM) learning algorithm was used, with a threefold cross validation in a binary classification task. We used sliding window technique during the signal processing, and we also analysed the effect of window size for the analysis of each behaviour element to achieve the most effective settings. Seventeen activities out of 40 were successfully recognized with AUC values above 0.8.

In summary, the LGBM is a very promising solution for recognizing daily routine or playful activities among children in real life situations, which is not sensitive to the window size. Big advantage of our finding that this machine learning method even works on commercially available devices, which could open the window for more promising examination of children behaviour in every-day situations.
